# Ganglioside composition and histology of a spontaneous metastatic brain tumour in the VM mouse

**DOI:** 10.1054/bjoc.2001.1909

**Published:** 2001-07

**Authors:** M El-Abbadi, T N Seyfried, A J Yates, C Orosz, M C Lee

**Affiliations:** Department of Biology, Boston College, Chestnut Hill, MA, 02467, USA; Department of Pathology, Chonnam National University Medical School, Kwangju, Korea; Division of Neuropathology, The Ohio State University, Columbus, OH, USA

**Keywords:** metastasis, cell culture, brain tumour, sialic acid, gangliosides, macrophages, VM mouse, neuraminic acid

## Abstract

Glycosphingolipid abnormalities have long been implicated in tumour malignancy and metastasis. Gangliosides are a family of sialic acid-containing glycosphingolipids that modulate cell–cell and cell–matrix interactions. Histology and ganglioside composition were examined in a natural brain tumour of the VM mouse strain. The tumour is distinguished from other metastatic tumour models because it arose spontaneously and metastasizes to several organs including brain and spinal cord after subcutaneous inoculation of tumour tissue in the flank. By electron microscopy, the tumour consisted of cells (15 to 20 μm in diameter) that had slightly indented nuclei and scant cytoplasm. The presence of smooth membranes with an absence of junctional complexes was a characteristic ultrastructural feature. No positive immunostaining was found for glial or neuronal markers. The total ganglioside sialic acid content of the subcutaneously grown tumour was low (12.6 ± 0.9 μg per 100 mg dry wt, *n*= 6 separate tumours) and about 70% of this was in the form of N-glycolylneuraminic acid. In contrast, the ganglioside content of the cultured VM tumour cells was high (248.4 ± 4.4 μg, *n*= 3) and consisted almost exclusively of N-acetylneuraminic acid. The ganglioside pattern of the tumour grown subcutaneously was complex, while GM3, GM2, GM1, and GD1a were the major gangliosides in the cultured tumour cells. This tumour will be a useful natural model for evaluating the role of gangliosides and other glycolipids in tumour cell invasion and metastasis. © 2001 Cancer Research Campaign http://www.bjcancer.com


British Journal of Cancer (2001) 85(2), 285–292
© 2001 Cancer Research Campaign
doi: 10.1054/ bjoc.2001.1909, available online at http://www.idealibrary.com on IDEAL®

http://www.bjcancer.com

### Ganglioside composition and histology of a spontaneous metastatic brain tumour in the VM mouse

**M El-Abbadi[1], TN Seyfried[1], AJ Yates[3], C Orosz[3] and MC Lee[2]**

[1]Department of Biology, Boston College, Chestnut Hill, MA 02467, USA; [2]Department of Pathology, Chonnam National University Medical School, Kwangju, Korea; [3]Division of Neuropathology, The Ohio State University, Columbus OH, USA

**Summary** Glycosphingolipid abnormalities have long been implicated in tumour malignancy and metastasis. Gangliosides are a family of sialic acid-containing glycosphingolipids that modulate cell–cell and cell–matrix interactions. Histology and ganglioside composition were examined in a natural brain tumour of the VM mouse strain. The tumour is distinguished from other metastatic tumour models because it arose spontaneously and metastasizes to several organs including brain and spinal cord after subcutaneous inoculation of tumour tissue in the flank. By electron microscopy, the tumour consisted of cells (15 to 20 µm in diameter) that had slightly indented nuclei and scant cytoplasm. The presence of smooth membranes with an absence of junctional complexes was a characteristic ultrastructural feature. No positive immunostaining was found for glial or neuronal markers. The total ganglioside sialic acid content of the subcutaneously grown tumour was low (12.6 ± 0.9 µg per 100 mg dry wt, $n = 6$ separate tumours) and about 70% of this was in the form of N-glycolylneuraminic acid. In contrast, the ganglioside content of the cultured VM tumour cells was high (248.4 ± 4.4 µg, $n = 3$) and consisted almost exclusively of N-acetylneuraminic acid. The ganglioside pattern of the tumour grown subcutaneously was complex, while GM3, GM2, GM1, and GD1a were the major gangliosides in the cultured tumour cells. This tumour will be a useful natural model for evaluating the role of gangliosides and other glycolipids in tumour cell invasion and metastasis. © 2001 Cancer Research Campaign http://www.bjcancer.com

**Keywords**: metastasis; cell culture; brain tumour; sialic acid; gangliosides; macrophages; VM mouse; neuraminic acid

Cancer metastasis is a complex problem that involves intrinsic properties of the metastatic tumour cells as well as interactions between these cells and the host immune system (Nicolson, 1984; Kanda et al, 1992). Metastatic tumour cells are distinguished from nonmetastatic tumour cells by their ability to move from a primary tumour site to a distant location where they adhere and grow. Alterations in the cell surface glycocalyx may underlie the metastatic potential of some tumour cells (Kim et al, 1975; Hakomori, 1996).

Gangliosides are sialic acid-containing glycosphingolipids (GSLs) that are enriched in the outer leaflet of plasma membranes. Marked changes occur in the content and distribution of gangliosides in association with brain tumour formation in both man and mouse (Seyfried et al, 1987; 1992; Fredman, 1988; Yates, 1988; Sung et al, 1994, 1995). Some of these changes are intrinsic to the neoplastic tumour cells and others are associated with the invasion of tumour-infiltrating host cells (El-Abbadi and Seyfried, 1994; Seyfried et al, 1996). Because gangliosides are major components of the cell surface glycocalyx and participate in cell–cell and cell–matrix interactions, they have been implicated in the invasive/metastatic properties of tumour cells (Kanda et al, 1992; Coulombe and Pelletier, 1993; Zebda et al, 1995; Hakomori, 1996; Bai and Seyfried, 1997; Fang et al, 1997; Ruggieri et al, 1999; Deng et al, 2000).

In this study, we analysed the ganglioside composition of a spontaneous malignant brain tumour in the VM mouse. Spontaneous tumours of the central nervous system (CNS) are rare in mice and occur with an overall incidence of about 0.01% (Swenberg, 1982; Morgan et al, 1984). The VM mouse strain is unique in this regard by expressing a high incidence (about 1.5%) of spontaneous CNS tumours that appear histologically as astrocytomas (Fraser, 1971, 1986). The VM brain tumour described here is highly metastatic and its ganglioside content is significantly influenced by in vivo and in vitro growth environments.

*Received 28 September 2000*
*Revised 24 April 2001*
*Accepted 26 April 2001*

*Correspondence to:* TN Seyfried

### MATERIALS AND METHODS

### Mice

VM mice were obtained originally as a gift from Dr George Carlson, McLaughlin Research Institute, Montana. All VM mice used in this study were propagated in the animal room of the Biology Department, Boston College, using animal husbandry conditions described previously (Flavin et al, 1991). A 425-day-old mouse from this colony showed enlargement of the head, lethargy and tremor. This mouse was killed by cervical dislocation and a tumour was grossly identified in a cerebral hemisphere showing a poorly defined 3 × 1 × 1 mm mass with regions of softening. The tumour was minced, suspended in phosphate-buffered saline (PBS), and was then implanted intracranially (i.c.) into 3 syngeneic VM mice. After 3 i.c. passages of the original tumour, serial subcutaneous (s.c.) transplants into the flank were initiated as described previously (El-Abbadi and Seyfried, 1994). Flank tumours became grossly evident within 8–10 days of transplantation, and death usually occurred within 4–6 weeks. VM mice, approximately 2–3 months of age, were used as tumour recipients. All animal use procedures were in accordance with the National

Institutes of Health Guide for the Care and Use of Laboratory Animals and were approved by the Institutional Care Committee. Also, these procedures meet the standards required by the UKCCCR guidelines.

### Histological analysis

Complete autopsies were performed to characterize the VM tumour and its metastatic involvement at 16, 20 and 30 days after flank implantation of the tumour tissue. Routine haematoxylin and eosin (H&E) staining was used to assess the histological appearance of primary and metastatic lesions. Immunohistochemical techniques were performed on both fresh-frozen and paraffin-embedded tumour tissues using the standard 3-step indirect peroxidase-antiperoxidase method (Sternberger, 1979). Primary antibodies were used for immunohistological analysis of the following proteins (a) neuroectodermal S-100 protein (BioGenex) (Kahn et al, 1983); (b) neuron-specific enolase (NSE) (Vinores et al, 1984); (c) neurofibrillary protein (NFP) (BioGenex) (Schlaepfer, 1987); (d) glial fibrillary acidic protein (GFAP) (Eng, 1985); (e) leukocyte common antigen (LCA) (Dako and ATCC) (Powers et al, 1992); (f) Mac-1α (Sanchez Madrid et al, 1983); (g) B cell surface glycoprotein (B 220) (Coffman and Weissman, 1981); and (h) intercellular adhesion molecule (ICAM-1) (Said et al, 1979). The antibodies were titrated against known positive tissues to determine the optimal concentrations to be used. For ultrastructural examination, the tumour tissue was fixed for 2 h at room temperature with 2.5% glutaraldehyde in 0.2 M cacodylate buffer (pH 7.2). Following post fixation in 2% osmium tetroxide for one hour at 4˚C, the tissue was dehydrated and embedded in an epon mixture, sectioned, and stained in uranyl acetate and lead citrate for 15 min and 10 min, respectively. Tissue sections were examined using a Philips 300 electron microscope.

### Ganglioside analysis

The solid tumours, normal mouse brains, and cultured tumour cells were frozen at –20˚C, and were then lyophilized to remove water. Gangliosides were isolated and purified by our previously described methods (Seyfried et al, 1978, 1987). The purified gangliosides were treated with mild base and then desalted using C-18 Bond Elute columns. Quantification of N-acetylneuraminic acid (NeuAc) and N-glycolylneuraminic acid (NeuGc) was determined using a gas-liquid chromatography procedure (Yu and Ledeen, 1970). Sialic acid concentration was expressed as μg 100 $mg^{-1}$ dry weight. The distribution of tumour gangliosides was examined using high performance thin-layer chromatography (HPTLC) (Whatman HPK silica gel) according to the method of Ando et al, 1978. The mouse brain tumour gangliosides were compared with gangliosides isolated from the cerebral cortex of normal adult mice and with purified GM3, GM3-NeuGc, GM2, GM2-NeuGc, GM1, GD3, GD1a, GT1a, GD1b, GT1b, and GQ1b isolated from mouse, bovine and human tissues by the method of Ando and Yu (Ando and Yu, 1977, 1979). The conditions for HPTLC development are described in Figure 3.

### Ganglioside biosynthesis in cultured tumour cells

The VM tumour cell line was established from the flank growing tumour as previously described for other mouse brain tumours (Seyfried et al, 1992; El-Abbadi and Seyfried, 1994). Ganglioside studies were conducted on confluent cell cultures that had a minimum of 10 passages. The cells were grown in T-75 cultured flasks until preconfluency. An aliquot of $^{14}$C-galactose (New England Nuclear) equivalent to 3–5 μCi was evaporated to dryness under $N_2$ and resuspended in 10 ml of complete Dulbecco's modified Eagle medium (DMEM). After passage through a sterile filter, the radiolabel-containing medium was fed to the cells. The cells were grown for 48 h (confluency stage) at 37˚C. The medium containing $^{14}$C-galactose was discarded and the flask rinsed 3 × with 5 ml of sterile, cold PBS. Next, the cells were trypsinized, collected in cold, sterile PBS, and centrifuged for 3 min at 2000 rpm. The pellet was washed twice with PBS, frozen, and finally lyophilized to dryness. Radiolabled gangliosides, synthesized by the cells, were isolated and purified as described above. Prior to ganglioside isolation, about 10 μg of unlabelled mouse brain ganglioside sialic acid were added to each sample as carrier. Approximately 3800 dpm of $^{14}$C-labelled gangliosides were spotted on the HPTLC plate.

Gangliosides from the VM cell line were treated with *Clostridium perfringens* neuraminidase (Type V). Neuraminidase removes terminal sialic residues from gangliosides and will convert hematosides (GM3 and GD3) to lactosylceramide, and polysialogangliosides to GM1 (Ando and Yu, 1977). Gangliosides GM1 and GM2 are mostly unaffected by neuraminidase treatment since they carry an internal sialic acid. The enzymatic hydrolysis was done as previously described (Ando and Yu, 1977). An aliquot of 5800 dpm of the radiolabelled purified tumour cell gangliosides was dried under $N_2$, then dissolved in 140 μl of 0.1 M sodium acetate buffer (pH 5.0). Next, 50 μl of neuraminidase (1 unit $ml^{-1}$ buffer) and 15 μl of 2% Triton CF-54 were added and the mixture was incubated for 24 hours at 37˚C in a shaking water bath. The reaction was stopped by boiling the mixture for 4 minutes. The reaction product was treated with mild base (0.1 N NaOH), desalted, resuspended in C:M (1:1) and finally spotted on HPTLC. Autoradiography of labelled gangliosides was performed by exposing the HPTLC plate to Hyperfilm-$^{3}$H (Amersham, Arlington Heights, IL) for 14 days at room temperature. The exposed film was then developed for 5 min and fixed for 4 minutes in Kodak GBX developer and fixer.

### RESULTS

### Histological analysis

Gross examination of the flank implanted tumours disclosed a whitish, solid, lobulated mass with areas of necrosis. Metastases of the tumour to brain, liver, spleen, and regional lymph were noted at the complete autopsy. Metastases to other organs were identified only by histologic examination. The most common and earliest sites to become involved with metastatic tumour were the brain, spleen, regional lymph nodes, vertebrae and spinal cord. Brain metastases were malignant and metastatic when transplanted subcutaneously into the flanks of other VM mice. Interestingly, no metastases occurred if the tumour was maintained through intracranial transplantation. The gross autopsy findings are summarized in Table 1.

By light microscopy, the tumour was diffusely infiltrating, highly cellular, and had a relatively uniform appearance (Figure 1A). The tumour cells had small amounts of cytoplasm, were poorly differentiated, and were supported by a delicate fibrovascular stroma. The tumour cell nuclei were centrally located, round

 © 2001 Cancer Research Campaign

**Table 1** Metastatic involvement of the spontaneous VM tumour

| Duration of tumour growth (days) | Tumour weight (g) | Tissues examined[a]: Lymph node | Skeletal muscle | Heart | Lung | Liver | Spleen | Kidney | Brain | Spinal cord |
|---|---|---|---|---|---|---|---|---|---|---|
| 16 | 0.30 | – | – | – | – | – | – | – | – | – |
| | 0.39 | – | – | +[b] | – | – | + | + | + | – |
| | 0.37 | + | – | – | – | – | + | – | + | – |
| 20 | 0.51 | – | – | – | – | + | + | – | + | + |
| | 0.57 | + | – | – | – | – | + | + | + | + |
| | 0.61 | + | + | – | – | – | + | + | + | + |
| 30 | 4.27 | + | – | –[c] | –[c] | + | + | + | + | + |
| | 4.41 | + | + | – | + | + | + | – | + | + |
| | 4.50 | + | + | – | + | + | + | + | + | + |

[a] + and – indicate the presence or absence of tumour, respectively. [b] Pericardial metastasis was noted. [c] Intravascular tumour emboli were detected.

or slightly cleaved, and contained coarse chromatin with prominent nucleoli. Mitotic figures and regions of necrosis were frequently seen, but nuclear pleomorphism was mild. The histologic features of the metastatic tumour foci in other organs were basically the same as those of the flank tumours. All but one animal had brain involvement with multiple foci of metastases consisting of diffuse infiltration of superficial cortex, perivascular Virchow-Robin's spaces of the grey and white matter, and subependymal regions (Figure 1B and C). No metastases to the vertebrae and spinal cord were seen after 16 days, but were present in all animals 20 and 30 days after flank implantation. In affected animals there was infiltration of tumour cells into dura mater of the spinal cord and peripheral nerve root (Figure 1D).

Organomegaly at autopsy identified early tumour involvement in regional lymph nodes and spleen. Metastases to liver and lung occurred later. Involvement of kidney was sporadic, occurring in at least 1 of 3 animals at each time following implantation. Microscopic examination disclosed multiple subcapsular metastases of the liver and diffuse infiltration of tumour cells into the sinusoids of the regional lymph nodes and spleen. Subcapsular and cortical metastases were noted in the kidney. The tumour involved the muscle of the flank near the implantation site suggesting direct invasion of the tumour cells into neighbouring muscle. The tumour cells also infiltrated into the subcutaneous fatty tissue of the flank. A pericardial metastasis was noted in one animal.

Staining for all neuroectodermal, neuronal, and glial markers (S-100, NSE, NFP and GFAP) were negative in both the implanted flank tumours and in the secondary metastatic tumours. The lymphoid markers (LCA) were mostly negative. In the metastatic spleen, however, there was a weakly positive reaction against B-220 (B lymphocyte marker) and a few positive cells were seen with Mac-1α (macrophage marker) and with ICAM-1 (proliferating lymphocytes and endothelial cells (not shown).

By electron microscopy, the tumour consisted of cells (15 to 20 μm in diameter) that had regular, slightly indented nuclei (Figure 2A). These cells contained densely clumped heterochromatin, prominent nucleoli, and had scant cytoplasm with only a small number of cytoplasmic organelles (Figure 2A and 2B). Groups of tumours cells were surrounded by attenuated fibroblasts with rough endoplasmic reticulum and long fibrillary cytoplasmic processes that were interposed between the tumour cells. The processes mimicked subplasmalemmal densities (Figure 2B). Frequent mitotic figures were noted. No intermediate filaments, microtubules, neurosecretory granules or cell processes were seen. The tumour cells had smooth, closely apposed membranes with no specific junctional structures (Figure 2C).

### Ganglioside analysis

The ganglioside sialic acid content of the VM tumour and cultured VM tumour cells is shown in Table 2. The total ganglioside sialic acid content of the s.c. and i.c. VM tumours was significantly lower than the content of the normal VM mouse brain (337.1 ± 10.5 μg NeuAc/100 mg dry weight; $n$ = 3). No NeuGc containing gangliosides were found in the brain. The ganglioside content in the s.c. tumour was very low and consisted mostly of NeuGc-containing gangliosides. The total sialic acid content was higher in the i.c. tumour than in s.c. tumour. This was due to a significant increase in NeuAc-containing gangliosides. This increase comes from the contamination of the tumour tissue with ganglioside-rich tissue from normal brain as we previously described for other mouse brain tumours (Seyfried et al, 1987; El-Abbadi and Seyfried, 1994). Normal neural tissue also contributes to the higher NeuAc ganglioside content in the brain metastasis (brain met.) than in s.c. tumour.

The i.c. tumour and the brain met. contained several gangliosides that co-migrated on HPTLC with mouse brain gangliosides (compare lanes 3, 4 and 6 in Figure 3). Several of these gangliosides (GD1a, GD1b, GT1b, and GQ1b) were absent or reduced in the s.c. tumour (lane 5). Most of these brain gangliosides originate from mouse brain tissue surrounding or entrapped within the tumour (Seyfried et al, 1987; El-Abbadi and Seyfried, 1994). The ganglioside patterns of the i.c. tumour and the brain met. were similar (lanes 4 and 6, respectively). Furthermore, these patterns represented a composite of the brain pattern (lane 3) and the s.c. tumour pattern (lane 5). The ganglioside pattern of the s.c. tumour was complex and contained several gangliosides that did not migrate on HPTLC with standard brain gangliosides (lane 5). A prominent band was seen migrating in the region of GM1b and multiple bands migrated in the region of GD1a/GT1a.

Striking differences in ganglioside content were found between the VM tumour grown in vivo and in vitro. The total ganglioside content and the content of NeuAc-containing gangliosides was about 20-fold higher and 70-fold higher, respectively, in the

© 2001 Cancer Research Campaign

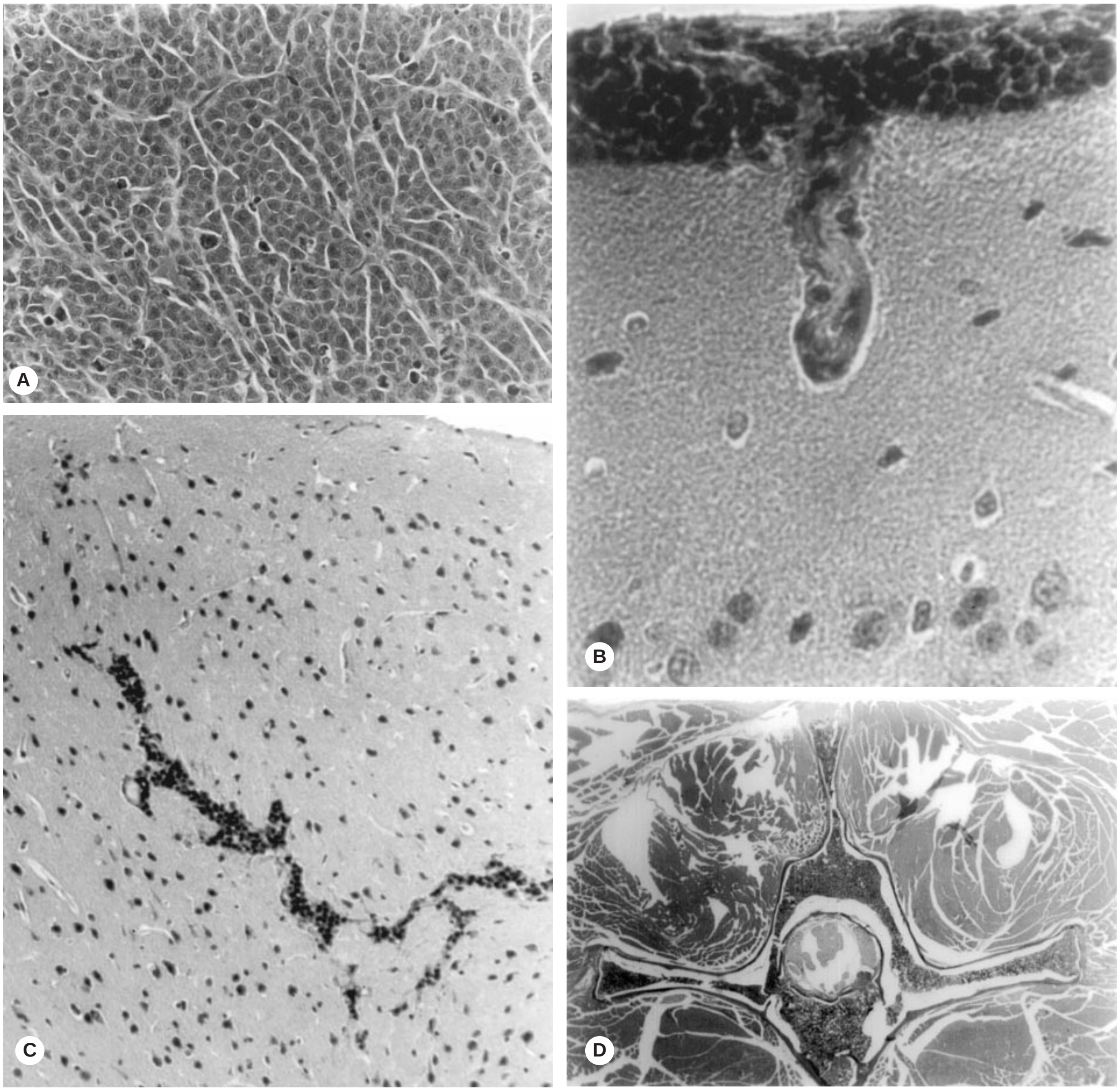

**Figure 1** Light microscopic appearance of the VM tumour 20 days after flank (s.c.) implantation. (**A**) The s.c. tumour consists of poorly differentiated, uniform, round cells with scant cytoplasm (H&E × 400). (**B–D**) Metastases from the s.c. VM tumour. (**B**) Metastatic tumour cells in the arachnoid (H&E × 400). (**C**) Metastatic tumour cells in frontal lobe (H&E × 100). (**D**) Metastatic tumour cells to vertebrae and arachnoid of the spinal cord (H&E, × 20)

cultured cells than in the s.c. tumour (Table 2). In contrast to the s.c. tumour, no NeuGc-containing gangliosides were detected in the cultured VM cells. GM3-NeuAc, GM2-NeuAc, GM1 (minor ganglioside), and GD1a were the predominant gangliosides in the VM cultured cells (Figure 4, lane 4). The same gangliosides were radiolabelled when the cells were grown in media containing $^{14}$C-galactose (Figure 4, lane 5). The ganglioside pattern in the cultured VM cells was less complex than the pattern observed in the tumour grown in vivo (compare lane 5, Figure 3 with lane 4, Figure 4).

The products of neuraminidase treatment of the radiolabelled gangliosides isolated from cultured VM cells is shown in Figure 4 (lane 6). Neuraminidase treatment converted several gangliosides to more simple structures. Specifically, the hydrolysis resulted in appearance of a double band in the lactosyl ceramide region, the disappearance of the double bands migrating with gangliosides GM3-NeuAc and GD1a, and an increase in the intensity of the double band migrating with GM1. These results indicate that GM3, GM2, GM1 and GD1a are the major gangliosides synthesized by the cultured VM tumour cells. Furthermore, the double band appearance of these gangliosides arises from heterogeneity in ceramide structure as described previously (Seyfried et al, 1987; El-Abbadi and Seyfried, 1994; Bai and Seyfried, 1997). A minor double band appeared above GM3 that migrated with standard GA2 (lane 6). The presence of Triton CF-54 in the neuraminidase reaction allows the neuraminidase to partially hydrolyse the internal sialic acid of GM2, thus producing GA2 (Li et al, 1980).

 *© 2001 Cancer Research Campaign*

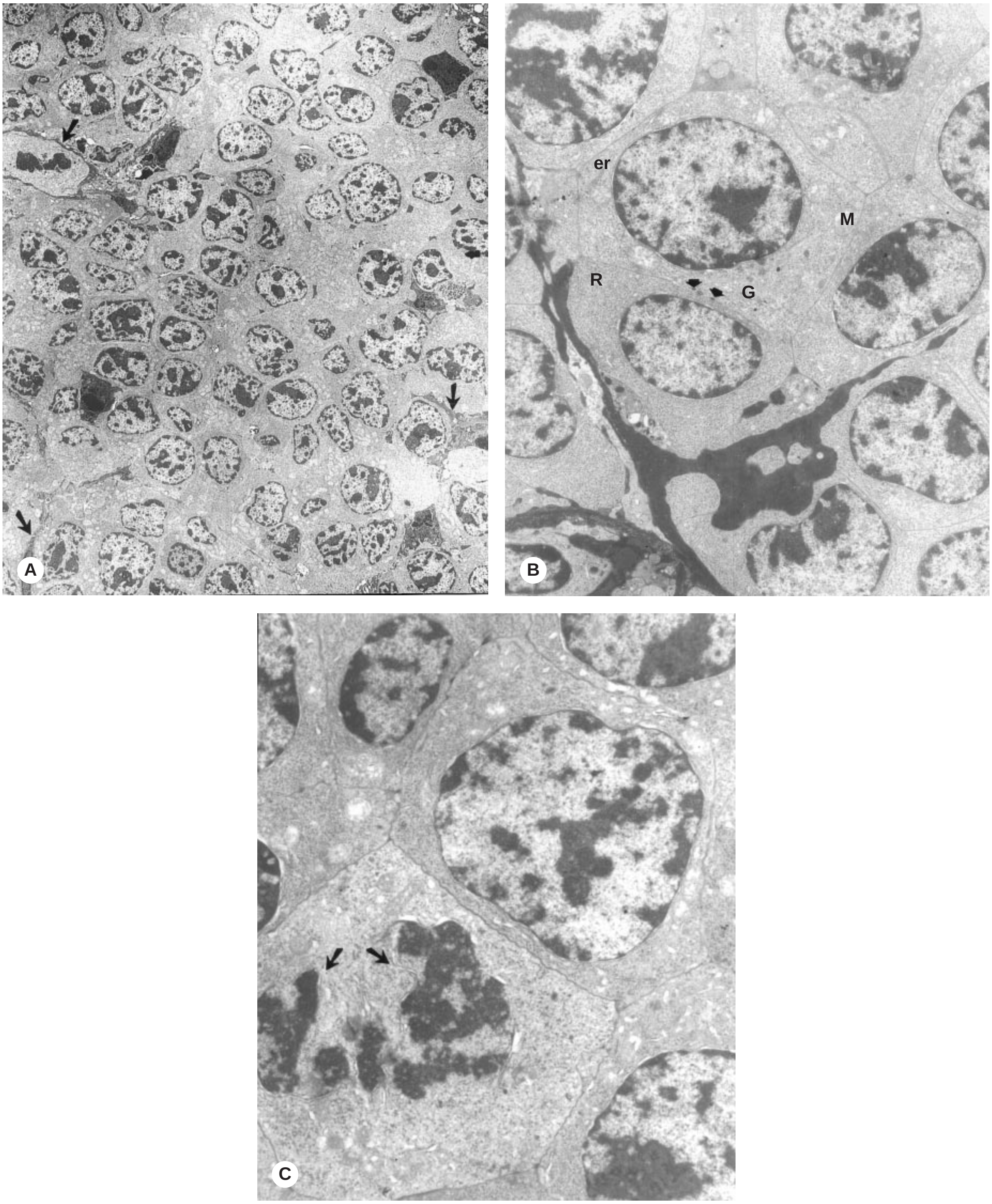

**Figure 2** Electron microscopic appearance of the VM tumour 20 days after flank (s.c.) implantation. (**A**) Medium-sized tumour cells (15–20 mM) surrounded by attenuated fibroblasts which have elongated fibrillary cytoplasmic processes (arrow) (× 2800); (**B**) Tumour cells have few mitochondria (M), single ribosomes (R), scant endoplasmic reticulum (er) and have closely apposed smooth membranes (× 12 800). The arrows indicate small, discrete interposition of fibrillary cytoplasmic processes between the tumour cells that mimic subplasmalemmal densities. (**C**) The tumour cells maintain their smooth outlines. There are no intermediate filaments, microtubules, neurosecretory granules, or cell processes. A nucleus undergoing mitosis is noted (arrows) (× 38 400)

### DISCUSSION

We have characterized the histology and ganglioside composition of a new and highly malignant brain tumour in the VM mouse strain. The tumour is unique because it arose spontaneously and metastasized to several organ systems including brain and spinal cord after simple s.c. inoculation of tumour tissue. Extensive metastasis distinguishes this VM brain tumour from other described VM mouse brain tumours and from most other metastatic murine tumour models that often require repeated in

**Table 2** Ganglioside content of the spontaneous metastatic VM brain tumour and cultured VM tumour cells

| VM tumour | N[b] | Ganglioside neuraminic acid content[a] (μg 100 mg$^{-1}$ dry wt) Total | NeuAc | NeuGc |
|---|---|---|---|---|
| Solid tumour | | | | |
| S.C.[c] | 6 | 12.6 ± 0.9 | 3.6 ± 0.6 | 8.9 ± 0.8 |
| I.C.[d] | 3 | 30.5 ± 7.9 | 24.6 ± 7.7 | 5.9 ± 0.3 |
| Brain Met.[e] | 3 | 15.5 ± 1.8 | 8.1 ± 2.0 | 7.5 ± 0.4 |
| Cultured cells[f] | 3 | 248.4 ± 4.4 | 248.4 ± 4.4 | ND[g] |

[a] NeuAc, *N*-acetylneuraminic acid; NeuGc, *N*-glycolylneuraminic acid. [b] N, Number of independent samples studied. Values are expressed as means ± SEM. [c] S.C., subcutaneously grown tumour.[d] I.C., intracerebrally grown tumour. [e] Brain Met., brain metasteses collected from mice carrying VM tumour in the flank. [f] Cultured VM cells were derived from the solid VM tumour as described in the Materials and Methods. [g] ND, not detectable.

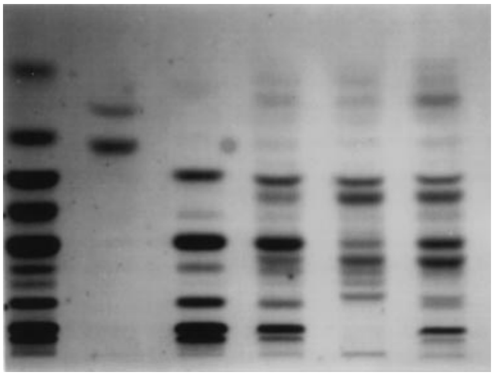

**Figure 3** Thin-layer chromatogram of VM tumour gangliosides from tumours grown in VM mice. **Lane 1**, purified ganglioside standards. GT1a and disialoparagloboside (LD1) co-migrate in this solvent system; **lane 2**, GM3-NeuGc and GM2-NeuGc standards; **lane 3**, adult VM mouse brain gangliosides; **lane 4**, gangliosides from tumour grown intracerebrally; **lane 5**, gangliosides from tumour grown subcutaneously in the flank; **lane 6**, gangliosides from brain metastases in mice carrying the tumour in the flank. The plate was developed by one ascending run with chloroform: methanol: water (55:45:10 by volume) that contained 0.02% $CaCl_2$:$2H_2O$. Approximately 2.0 μg of total ganglioside sialic acid were spotted onto each lane. After development, the bands were visualized by the resorcinol spray

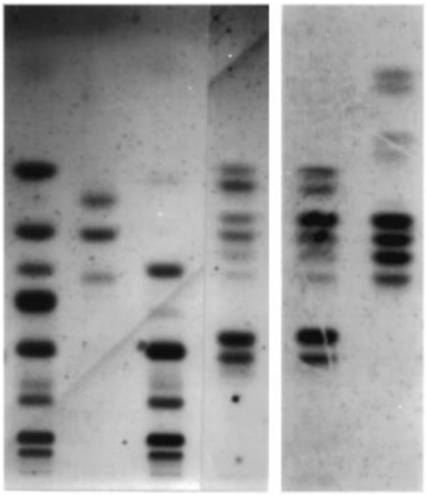

**Figure 4** Thin-layer chromatogram of gangliosides in cultured VM tumour cells. **Lane 1**, ganglioside standards; **lane 2**, GM3-NeuGc and GM2-NeuGc and GM1b standards; **lane 3**, adult VM brain gangliosides; **lane 4**, gangliosides in cultured VM tumour cells; **lane 5**, autoradiogram of radiolabelled gangliosides synthesized by cultured VM cells; **lane 6**, autoradiogram of neuraminidase-treated gangliosides synthesized by cultured VM cells. For radiolabelling, cells were grown for 48 h in media containing $^{14}$C-galactose. After isolation and purification of labelled tumour cell gangliosides, an aliquot containing about 1900 dpm was spotted onto the HPTLC plate (**lane 5**). For *C. perfringens* neuraminidase treated samples (**lane 6**), an aliquot containing about 5400 dpm was spotted onto HPTLC plate. Approximately 2.0 μg of total ganglioside sialic acid were spotted for each of unlabelled lanes (**lane 1–4**). The plate was developed by one ascending run with chloroform:methanol:water that contained 0.02% $CaCl_2$:$2H_2O$; (55:45:10 by volume). The dried plates were exposed to Hyperfilm-$^3$H. After autoradiography, the plates were sprayed with the resorcinol reagent to identify lanes with unlabelled gangliosides vivo selection of brain colonizing variants from tumour cell lines that routinely metastasize to nonneural organs (Bosmann et al, 1973; Brunson et al, 1978; Conley, 1984; Schackert et al, 1988). Hence, this new VM tumour should be useful as a natural model for human metastatic cancer.

Although this VM tumour is highly metastatic when implanted subcutaneously, it does not metastastize to non-CNS organs when implanted into the brain. Extracranial metastasis is also generally rare for most primary human brain tumours (Duffy, 1983; Shapiro, 1986; Pedersen et al, 1994; Vural et al, 1996). The explanation for this is unknown, but it may be related to the immunologically privilaged nature of the brain or to the inability of VM cells to adhere and/or degrade brain vascular endothelium. The VM brain tumour may therefore serve as a model for investigating these processes.

The poorly differentiated appearance the VM tumour cells under light microscopy is consistent with previous observations in other VM tumours (Serano et al, 1980; Pilkington et al, 1985). Although the VM brain tumours described by H Fraser appeared histologically as astrocytomas (Fraser, 1971, 1986), it was not possible for us to classify the metastatic VM brain tumour as an astrocytoma since no positive staining was seen with GFAP, NSE, NFP or S-100 protein. It is important to mention, however, that Fraser examined the histology of primary VM tumours at their site of origin, while we examined the histology of our VM tumour following serial passage. This could account in part for some of the histological differences between studies on VM tumours since tumour microenvironment can influence tumour histology

 © 2001 Cancer Research Campaign

(Seyfried, 2001). Our electron microscopic examination also revealed a poorly differentiated tumour with a sparse interstitial matrix and closely apposed cell surface. Based on the weak immunostaining of the VM tumour with B220, we cannot exclude the possibility of lymphoma. Further studies will be needed to better classify this tumour.

The ganglioside content of this tumour was different from that of previously described mouse brain tumours. In contrast to most experimental mouse brain tumours, which contain between 20–70 μg total ganglioside sialic acid/100 mg dry weight when grown subcutaneously in vivo (Seyfried et al, 1987, 1992), the total ganglioside sialic acid content of the VM tumour was only about 13 μg. Furthermore, about 70% of this sialic acid was in the form of NeuGc. Our previous studies showed that much of the NeuGc-containing gangliosides found in mouse brain tumours is contributed by the host environment, e.g., serum and tumour-infiltrating macrophages (Ecsedy et al, 1998; El-Abbadi and Seyfried, 1994; Seyfried et al, 1996, 1998). Our findings suggest that the total ganglioside content of the neoplastic VM tumour cells is diminished when grown in vivo.

Differences in growth environment (in vivo versus in vitro) significantly influenced the ganglioside content of the metastatic VM tumour. In contrast to the in vivo-grown VM tumour, which contained a low total ganglioside content, the ganglioside sialic acid content of the cultured VM tumour cells was high (Table 2). Indeed, this content was much higher than that found previously in 20-methylcholanthrene-induced experimental mouse brain tumour cell lines (about 38–82 μg 100 $mg^{-1}$ dry weight) (Seyfried et al, 1992). Muthing and coworkers previously reported that differences in the in vitro growth environment could significantly influence the ganglioside composition of mouse lymphoma cells (Muthing et al, 1992). Differences in growth environment can also influence the ganglioside distribution of human brain tumours (Wikstrand et al, 1994; Fredman et al, 1996).

It is unlikely that the low ganglioside content of the in vivo-grown VM tumour results from a tumour-shedding phenomenon since serum ganglioside content is lower in tumour-bearing VM mice than in nontumour-bearing VM mice (Cotterchio and Seyfried, 1994). This contrasts with previous findings in the murine AKR lymphoma and ependymoblastoma which shed gangliosides (Ladisch et al, 1987; Manfredi et al, 1999). Since most gangliosides in serum are derived from the liver, we previously suggested that the low serum ganglioside content in the tumour-bearing VM mice may result from impaired liver function due to liver metastasis (Cotterchio and Seyfried, 1994). The presence of VM tumour cells in liver supports this possibility.

The low ganglioside content of the in vivo-grown VM tumour may be associated with its high metastatic potential. Support for this comes from previous studies that some metastatic tumours grown in vivo contain a lower total ganglioside content than their nonmetastatic variants (Kloppel et al, 1979; Merritt et al, 1980; Skipski et al, 1981; Niederkorn et al, 1993; Zebda et al, 1995). Also, an opposite effect is observed for cultured tumour cells where the ganglioside content is generally higher in the metastatic than the nonmetastatic cell lines (Yogeeswaran and Salk, 1981; Yogeeswaran, 1983; Laferte et al, 1987). Our findings on the metastatic VM brain tumour model are consistent with these observations. Additionally, Ladisch and co-workers recently reported a positive correlation between the ganglioside content of murine melanoma cell lines and the metastatic potential of resulting in vivo tumours (Deng et al, 2000). The VM tumour may therefore serve as a model for evaluating the role of gangliosides and other glycolipids in tumour cell invasion and metastasis.

### ACKNOWLEDGEMENTS

This research was supported by NIH grants CA33640 (TNS) and CA50910 (AJY), and from the Boston College Research Expense Fund. We thank Jeffrey Ecsedy for technical support.

### REFERENCES

Ando S and Yu RK (1977) Isolation and characterization of a novel trisialoganglioside, GT1a, from human brain. *J Biol Chem* **252**: 6247–6250

Ando S and Yu RK (1979) Isolation and characterization of two isomers of brain tetrasialogangliosides. *J Biol Chem* **254**: 12224–12229

Bai H and Seyfried TN (1997) Influence of ganglioside GM3 and high density lipoprotein (HDL) on the cohesion of mouse brain tumor cells. *J Lipid Res* **38**: 160–172

Bosmann HB, Bieber GF, Brown AE, Case KR, Gersten DM, Kimmerer TW and Lione A (1973) Biochemical parameters correlated with tumour cell implantation. *Nature* **246**: 487–489

Brunson KW, Beattie G and Nicolsin GL (1978) Selection and altered properties of brain-colonising metastatic melanoma. *Nature* **272**: 543–545

Coffman RL and Weissman IL (1981) B220: a B cell-specific member of the T200 glycoprotein family. *Nature* **289**: 681–683

Conley FK (1984) Metastatic brain tumor model in mice that mimics the neoplastic cascade in humans. *Neurosurgery* **14**: 187–192

Cotterchio M and Seyfried TN (1994) Serum gangliosides in mice with metastatic and non-metastatic brain tumors. *J Lipid Res* **35**: 10–14

Coulombe J and Pelletier G (1993) Gangliosides and organ-specific metastatic colonization. *Int J Cancer* **53**: 104–109

Deng W, Li R and Ladisch S (2000) Influence of cellular ganglioside depletion on tumor formation. *J Natl Cancer Inst* **92**: 912–917

Duffy PE (1983) *Astrocytes: Normal, Reactive, and Neoplastic*. Raven Press: New York

Ecsedy JA, Yohe HC, Bergeron AJ and Seyfried TN (1998) Tumour-infiltrating macrophages contribute to the glycosphinglipid composition of brain tumours. *J Lipid Res* **39**: 2218–2227

El-Abbadi M and Seyfried TN (1994) Influence of growth environment on the ganglioside composition of an experimental mouse brain tumor. *Mol Chem Neuropathol* **21**: 273–285

Eng LF (1985) Glial fibrillary acidic protein (GFAP): the major protein of glial intermediate filaments in differentiated astrocytes. *J Neuroimmunol* **8**: 203–214

Fang LH, Lucero M, Kazarian T, Wei Q, Luo FY and Valentino LA (1997) Effects of neuroblastoma tumor gangliosides on platelet adhesion to collagen. *Clin Exp Metastasis* **15**: 33–40

Flavin HJ, Wieraszko A and Seyfried TN (1991) Enhanced aspartate release from hippocampal slices of epileptic (El) mice. *J Neurochem* **56**: 1007–1011

Fraser H (1971) Astrocytomas in an inbred mouse strain. *J Pathol* **103**: 266–270

Fraser H (1986) Brain tumours in mice, with particular reference to astrocytoma. *Food Chem Toxicol* **24**: 105–111

Fredman P (1988) Gangliosides in human malignant gliomas. In *New Trends in Ganglioside research: Neurochemical and Neurogenerative Aspects* Ledeen RW, Hogan EL, Tettamanti G, Yates A and Yu RK (eds) pp. 151–161. Liviana: Padova

Fredman P, Wikstrand CJ, Mansson JE, Reifenberger G, Bigner SH, Rasheed A, Svennerholm L and Bigner DD (1996) In vivo growth conditions suppress the expression of ganglioside GM2 and favour that of lacto series gangliosides in the human glioma D-54MG cell line. *Glycoconj J* **13**: 391–399

Hakomori S (1996) Tumor malignancy defined by aberrant glycosylation and sphingo (glyco) lipid metabolism. *Cancer Res* **56**: 5309–5318

Kahn HJ, Marks A, Thom H and Baumal R (1983) Role of antibody to S100 protein in diagnostic pathology. *Am-J-Clin-Pathol* **79**: 341–347

Kanda S, Cochran AJ, Lee WR, Morton DL and Irie RF (1992) Variations in the ganglioside profile of uveal melanoma correlate with cytologic heterogeneity. *Int J Cancer* **52**: 682–687

Kim U, Baumler A, Carruthers C and Bielat K (1975) Immunological escape mechanism in spontaneously metastasizing mammary tumors. *Proc Natl Acad Sci USA* **72**: 1012–1016

Kloppel TM, Morre DJ and Jacobsen LB (1979) Ganglioside patterns of metastatic and non-metastatic transplantable hepatocellular carcinomas of the rat. *J Supramol Struct* **11**: 485–492

© 2001 Cancer Research Campaign 

Ladisch S, Kitada S and Hays EF (1987) Gangliosides shed by tumor cells enhance tumor formation in mice. *J Clin Invest* **79**: 1879–1882
Laferte S, Fukuda MN, Fukuda M, Dell A and Dennis JW (1987) Glycosphingolipids of lectin-resistant mutants of the highly metastatic mouse tumor cell line, MDAY-D2. *Cancer Res* **47**: 150–159
Li Y-T, King MJ and Li SC (1980) Enzymatic degradation of gangliosides *Adv Exp Med Biol* **125**: 93–104
Manfredi MG, Lim S, Claffey KP and Seyfried TN (1999) Gangliosides influence angiogenesis in an experimental mouse brain tumor. *Cancer Res* **59**: 5392–5397
Merritt WD, Morre DJ, Doak RL and Keenan TW (1980) Gangliosides of liver tumors induced by N-2 fluorenylacetamide III. Galactosyl and sialyl transferases in single carcinomas and nodules. *Cancer Biochem Biophys* **4**: 183–194
Morgan KT, Frith CH, Swenberg JA, McGrath JT, Zulch KJ and Crowder DM (1984) A morphologic classification of brain tumors found in several strains of mice. *J Natl Cancer Inst* **72**: 151–160
Muthing J, Portner A and Jager V (1992) Ganglioside alterations in YAC-1 cells cultivated in serum-supplemented and serum-free growth medium. *Glycoconj J* **9**: 265–273
Nicolson GL (1984) Cell surface molecules and tumor metastasis. Regulation of metastatic phenotypic diversity. *Exp Cell Res* **150**: 3–22
Niederkorn JY, Mellon J, Pidherney M, Mayhew E and Anand R (1993) Effect of anti-ganglioside antibodies on the metastatic spread of intraocular melanomas in a nude mouse model of human uveal melanoma. *Curr Eye Res* **12**: 347–358
Pedersen PH, Rucklidge GJ, Mork SJ, Terzis AJ, Engebraaten O, Lund Johansen M, Backlund EO, Laerum OD and Bjerkvig R (1994) Leptomeningeal tissue: a barrier against brain tumor cell invasion. *J Natl Cancer Inst* **86**: 1593–1599
Pilkington GJ, Darling JL, Lantos PL and Thomas DG (1985) Tumorigenicity of cell lines (VMDk) derived from a spontaneous murine astrocytoma. Histology, fine structure and immunocytochemistry of tumours. *J Neurol Sci* **71**: 145–164
Powers JM, Liu Y, Moser AB and Moser HW (1992) The inflammatory myelinopathy of adreno-leukodystrophy: cells, effector molecules, and pathogenetic implications. *J Neuropathol Exp Neurol* **51**: 630–643
Ruggieri S, Mugnai G, Mannini A, Calorini L, Fallani A, Barletta E, Mannori G and Cecconi O (1999) Lipid characteristics in metastatic cells. *Clin Exp Metastasis* **17**: 271–276
Said JW, Hargreaves HK and Pinkus GS (1979) Non-Hodgkin's lymphomas: an ultrastructural study correlating morphology with immunologic cell type. *Cancer* **44**: 504–528
Sanchez Madrid F, Simon P, Thompson S and Springer TA (1983) Mapping of antigenic and functional epitopes on the alpha- and beta-subunits of two related mouse glycoproteins involved in cell interactions, LFA-1 and Mac-1. *J Exp Med* **158**: 586–602
Schackert G, Simmons RD, Buzbee TM, Hume DA and Fidler IJ (1988) Macrophage infiltration into experimental brain metastases: occurrence through an intact blood-brain barrier. *J Natl Cancer Inst* **80**: 1027–1034
Schlaepfer WW (1987) Neurofilaments: structure, metabolism and implications in disease. *J Neuropathol Exp Neurol* **46**: 117–129
Serano RD, Pegram CN and Bigner DD (1980) Tumorigenic cell culture lines from a spontaneous VM/Dk murine astrocytoma (SMA). *Acta Neuropathol Berl* **51**: 53–64
Seyfried TN (2001) Perspectives on brain tumor formation involving macrophages, glia, and neural stem cells. *Persp Biol Med* **44**: 263–282
Seyfried TN, Glaser GH and Yu RK (1978) Cerebral, cerebellar, and brain stem gangliosides in mice susceptible to audiogenic seizures. *J Neurochem* **31**: 21–27
Seyfried TN, Yu RK, Saito M and Albert M (1987) Ganglioside composition of an experimental mouse brain tumor. *Cancer Res* **47**: 3538–3542
Seyfried TN, El-Abbadi M and Roy ML (1992) Ganglioside distribution in murine neural tumors. *Mol Chem Neuropathol* **17**: 147–167
Seyfried TN, El-Abbadi M, Ecsedy JA, Bai HW and Yohe H (1996) Influence of host cell infiltration on the glycolipid content of mouse brain tumors. *J Neurochem* **66**: 2026–2033
Seyfried TN, El-Abbadi M, Ecsedy JA, Griffin ME and Yohe HC (1998) Ganglioside composition of a mouse brain tumor grown in the severe combined immunodeficiency (SCID) mouse. *Mol Chem Neuropathol* **33**: 27–37
Shapiro WR (1986) Therapy of adult malignant brain tumors: what have the clinical trials taught us? *Semin Oncol* **13**: 38–45
Skipski VP, Carter SP, Terebus-Kekish OI, Podlaski FJJ, Peterson RH and Stock CC (1981) Ganglioside profiles of metastases and of metastasizing and nonmetastasizing rat primary mammary carcinomas *J Natl Cancer Inst* **67**: 1251–1258
Sternberger LA (1979) *Immunocytochemistry* Wiley: New York
Sung CC, Pearl DK, Coons SW, Scheithauer BW, Johnson PC and Yates AJ (1994) Gangliosides as diagnostic markers of human astrocytomas and primitive neuroectodermal tumors. *Cancer* **74**: 3010–3022
Sung CC, Pearl DK, Coons SW, Scheithauer BW, Johnson PC, Zheng M and Yates AJ (1995) Correlation of ganglioside patterns of primary brain tumors with survival. *Cancer* **75**: 851–859
Swenberg JA (1982) Neoplasms of the nervous system. In *The Mouse in Biomedical Research* Foster HI, Small JD and Fox JG (eds), Vol. IV. pp. 529–527. Academic Press: New York
Vinores SA, Bonnin JM, Rubinstein LJ and Marangos PJ (1984) Immunohistochemical demonstration of neuron-specific enolase in neoplasms of the CNS and other tissues. *Arch Pathol Lab Med* **108**: 536–540
Vural G, Hagmar B and Walaas L (1996) Extracranial metastasis of glioblastoma multiforme diagnosed by fine-needle aspiration: a report of two cases and a review of the literature. *Diagn Cytopathol* **15**: 60–65
Wikstrand CJ, Fredman P, McLendon RR, Svennerholm L and Bigner DD (1994) Altered expression of ganglioside phenotypes of human gliomas in vivo and in vitro. *Mol Chem Neuropathol* **21**: 129–138
Yates AJ (1988) Glycolipids and gliomas. A review. *Neurochem Pathol* **8**: 157–180
Yogeeswaran G (1983) Cell surface glycolipids and glycoproteins in malignant transformation. *Adv Cancer Res* **38**: 289–344
Yogeeswaran G and Salk PL (1981) Metastatic potential is positively correlated with cell surface sialylation of cultured murine tumor cell lines. *Science* **212**: 1514–1516
Yu RK and Ledeen RW (1970) Gas–liquid chromatographic assay of lipid-bound sialic acids: measurement of gangliosides in brain of several species. *J Lipid Res* **11**: 506–516
Zebda N, Pedron S, Rebbaa A, Portoukalian J and Berthier Vergnes O (1995) Deficiency of ganglioside biosynthesis in metastatic human melanoma cells: relevance of CMP-NeuAc:LacCer alpha 2–3 sialyltransferase (GM3 synthase). *FEBS Lett* **362**: 161–164

 *© 2001 Cancer Research Campaign*
